# Aryl Hydrocarbon Receptor-Signaling Regulates Early *Leishmania major*-Induced Cytokine Expression

**DOI:** 10.3389/fimmu.2019.02442

**Published:** 2019-10-15

**Authors:** Niels-Arne Münck, Johannes Roth, Cord Sunderkötter, Jan Ehrchen

**Affiliations:** ^1^Institute of Immunology, University of Münster, Münster, Germany; ^2^Department of Translational Dermatoinfectiology, University of Münster, Münster, Germany; ^3^Department of Dermatology, University of Münster, Münster, Germany; ^4^Department of Dermatology and Venereology, Martin Luther University of Halle-Wittenberg, Halle, Germany

**Keywords:** skin infection, leishmaniasis, AhR, macrophages, epidermis

## Abstract

The early inflammatory skin micromilieu affects resistance in experimental infection with *Leishmania major*. We pursue the concept that macrophages, which take up parasites during early infection, exert decisive influence on the inflammatory micromilieu after infection. In order to analyze their distinctive potential, we identified differentially regulated genes of murine granuloma macrophages (GMΦ) from resistant and susceptible mice after their infection with metacyclic *Leishmania major*. We found induction of several cytokines in GMΦ from both strains and a stronger upregulation of the transcription factor aryl hydrocarbon receptor (AhR) in GMΦ from resistant mice. Using both an AhR agonist and antagonist we demonstrated that AhR is involved in *Leishmania*-induced production of TNF in macrophages. *In vivo*, single local injection of an AhR agonist in early lesions of susceptible mice caused an increased induction of *Tnf* and other cytokines in the skin. Importantly, local agonist treatment led to a reduction of disease severity, reduced parasite loads and a weaker Th2 response. Our results demonstrate that local activation of AhR has a beneficial effect in experimental leishmaniasis.

## Introduction

Interaction of pathogens and innate immune cells is a crucial early event in infection. It triggers immediate defense mechanisms and initiates pathogen-specific acquired immunity. Pathogens, however, have evolved mechanisms to circumvent activation of the immune system. Skin infection with intracellular parasite *Leishmania (L.) major* is an excellent murine model system to analyze host-pathogen interaction (1). Early after infection with *L. major*, macrophages take up infectious metacyclic promastigotes which then transform intracellularly into amastigotes. The innate immune system is unable to clear the infection without activation by T-cells. Ag-specific Th1 cells and their production of Interferon-γ (IFNγ) are necessary to achieve classical activation of macrophages and NO-mediated parasite clearing in resistant C57BL/6 mice (2). Susceptible BALB/c mice develop a Th2-response characterized by IL4 and IL13 secretion and fail to clear the parasite. Th-cell priming takes place in draining lymph nodes (dLN), but can be influenced by the inflammatory milieu of the infected skin (3–6). We already demonstrated differences in the early infiltrate of phagocytes with earlier appearance of mature macrophages in resistant mice (5, 7). Subsequently, we have identified several differentially regulated cytokines expressed in infected skin from resistant and susceptible mice early during infection. By altering the expression of some of these cytokines in the early inflammatory skin milieu, e.g., IL-6 and CXCL11, we were able to significantly influence polarization of Th-cells (5, 6).

Macrophages are among the first cells to interact with parasites and are capable of sustained production of inflammatory mediators. We thus consider them a prime candidate to determine the early inflammatory micromilieu.

Infection of macrophages with *L. major* has been shown to induce cytokine secretion, upregulation of genes and activation of cellular signaling pathways like ERK1/2, p38MAPK and NF-kappa-B (8–12). On the other hand, suppression of gene expression and inhibition of NF-kappa-B-signaling were reported (10, 13). For example, *L. major* uptake by macrophages abrogates LPS-induced IL12 secretion (8, 14).

However, differential reaction of macrophages to *L. major* also depends on the origin or activation of investigated macrophages or subtype of monocytes (15–18).

*L. major*-induced IL12 suppression was more pronounced in peritoneal macrophages compared to bone marrow macrophages (19). Similarly, a comparison of inflammatory and resident peritoneal macrophages infected with *L. major* revealed that resident, but not inflammatory macrophages induced production of various cytokines and reactive oxygen species (20).

Therefore, since macrophage subtypes can be different in response to *L. major*, we wanted to analyze cells which most closely resemble skin macrophages. Such cutaneous macrophages, well-suited for our study, can be isolated in considerable numbers from non-immune polyacrylamide granulomas (8, 21).

Strain-specific differences in skin macrophages could affect the early inflammatory milieu. Belkaid et al. already compared IL12, IL6, and TNF secretion of granuloma-macrophages (GMΦ) from C57BL/6 and BALB/c mice infected with *L. major*, and found no differences. For a more global analysis however, we performed microarray analysis of GMΦ infected with metacyclic *L. major* promastigotes. We identified differentially regulated genes between infected GMΦ from BALB/c and C57BL/6 mice. Among those we found transcription factor aryl hydrocarbon receptor (AhR), an inductor of xenobiotic compound-degrading enzymes, deserving a closer look because of its expanding role in immunity, especially macrophage function (22–26). Most relevant to our setting, Climaco-Arvizu et al. found a role for AhR in the regulation of nitric oxide and arginase production in mouse macrophages. Peritoneal macrophages from *Ahr* knockout mice showed reduced NO production, but enhanced secretion of several cytokines like TNF and IL12 when polarized to a M1 subtype and stimulated with LPS (26). *Ahr* knockout macrophages were more susceptible to *in vitro Leishmania* infection. In a previous work, the same group also reported that *Ahr* knockout mice on a resistant background showed an exacerbated immune response characterized by lower numbers of regulatory T-cells that, while causing more severe inflammation at first, ultimately resulted in accelerated control of the parasite (24). As AhR is a transcriptional regulator implicated in resistance against *Leishmania* both *in vitro* in macrophages and *in vivo*, we chose to further analyze the role of AhR in early events determining resistance against *L. major*.

## Materials and Methods

### Experimental Leishmaniasis

Specific pathogen free mice from C57BL/6 and BALB/c strains were bred in the animal facility of the Department of Dermatology, Münster, or obtained from Charles River, Germany, and used at 8-12w of age. All experiments were approved according to the animal welfare laws of the Federal Republic of Germany by the animal welfare authority of the state of North Rhine- Westphalia, filed under reference 84-02.04.2015.A348.

*Leishmania* parasites of strain MHOM/IL/81/FE/BNI were grown in Schneider's *Drosophila* medium supplemented with 10% fetal calf serum (FCS), 2% human urine, 2% glutamine, and 1% penicillin-streptomycin at 25°C and 5% CO_2_.

Male BALB/c mice (5 per group) were infected in the left hind foot with 2×10^7^ stationary phase parasites in 20 μl PBS to allow a comparison to our previously published data on the early phase of experimental leishmaniasis (5, 6). Mice were sacrificed after 20 h for measurement of epidermal gene expression or after 11 d (early Th response), and 4 or 5 w for infection experiments (late Th response, parasite load). Foot swelling was measured weekly using a caliper with the uninfected foot serving as control. All experiments were repeated at least 5 times.

For simultaneous treatment with AhR ligands, 30 nmol of agonist ITE (2-(1*H*-Indol-3-ylcarbonyl)-4-thiazolecarboxylic acid methyl ester, Tocris Bioscience) in 1 μl DMSO or solvent control was included in the final volume.

Comparison of parasite dissemination was performed by limiting dilution assay of infected dLN using *Leishmania* growth medium as described previously (5, 18) by using Leishmania medium instead of slant blood agar. Briefly, foot skin and dLN were removed aseptically and homogenized in 5 ml Leishmania medium. Serial dilutions were carried out in quadruples (100 μl culture volume each) using 96-well tissue plates. After culture for 1 w, the highest dilution yielding growth of viable parasites was determined using a phase contrast microscope.

### Cytokine Assay

Cytokines from restimulated *L. major*-specific dLN cells were assessed using a mixed lymphocyte reaction as previously described (5). Briefly, the dLN from infected animals were mashed through a cell strainer in PBS, washed and transferred to uncoated 96-well U-bottom plates in RPMI 1640 containing 2 mM glutamine, 50 μM mercaptoethanol, and 10% FCS at 2 × 10^6^ cells/well. Cells were restimulated with 1 μl soluble Leishmania antigen prepared by repeated freeze/thaw cycling of 5 × 10^8^ stationary phase parasites in 1 ml PBS. After 5d at 37°C and 5% CO_2_, IFNγ and IL4 secretion were measured by cytometric bead assay using FlexSets by BD Bioscience (San Jose, California) according to the manufacturer's protocol.

### Granuloma Macrophages

GMΦ were recovered from polyacrylamide gel pouches as described in John et al. (21). In short, sterile polyacrylamide gel (BioGel P-100, Bio-Rad, Germany) was injected subcutaneously in two 1 ml portions on the back of the animal. The gel was recovered after 48 h, resuspended in PBS and given through a cell strainer and then washed. GMΦ were then left to adhere in petri dishes with 10 ml DMEM medium containing 20% L-929 cell (ATCC #CCL-1) supernatant and 10% FCS at 1 × 10^6^cells/ml for 24 h at 37°C and 7% CO_2_. Cells were incubated with 10 mM EDTA in PBS for 10 min at 37° and resuspended by repeated up and down-pipetting. Cells were then washed with PBS and resuspended in DMEM containing L-929-cell supernatant at 1 × 10^6^cells/ml and left to adhere overnight in 12-well plates. Metacyclic *L. major* promastigotes were prepared from stationary cultures. Briefly, stationary phase cultures are centrifuged several times to enrich metacyclic parasites in the supernatant by their density after which the remaining parasites are collected and run over a Ficoll gradient as described in more detail by Späth et al. (27). Macrophages were incubated with a 5 × MOI of metacyclic *L. major*. 1 μl of 30 mM solutions of AhR antagonist CH-223191 and agonist ITE in sterile DMSO per ml medium were added where indicated, with untreated groups receiving 1 μl of DMSO. TNF concentration of the supernatant was measured using the cytometric bead assay FlexSet for murine TNF by BD Bioscience (San Jose, California). Parasite phagocytosis was measured by FACS analysis of cells incubated with a 5 × MOI of fluorescein isothiocyanate (FITC) -stained parasites in the presence of DMSO, ITE and CH-223191 using the same medium and concentrations as above. The FITC-staining was performed as described earlier (18). For assessment of parasite killing, cells where then washed and incubated in the presence of 500U of rmIFNγ (PromoCell, Germany) for another 20 h. Production of NO was measured by photometric measurement of nitrite in supernatant at 560 nm using Griess reagent and a nitrite standard curve after 24 h preincubation of 5 × 10^5^ cells/ml with 500U of recombinant murine IFNγ) followed by further 24 h incubation with *L. major* in the presence of 500U/ml rmIFNγ as described in Ehrchen et al. (18).

### Human Blood Monocytes

Human monocytes were isolated from fresh human blood leukocyte reduction chambers of platelet apheresis sets from healthy, voluntary whole blood donations after informed consent of the donors according to the regulations of the blood bank of the University Hospital Münster by Pancoll (PAA Laboratories, Austria) and subsequent Percoll (GE Healthcare) gradient centrifugation as described previously (28). Purity of monocytes was > 85%, as assessed by staining with CD14 antibody (Becton Dickinson) and FACS analysis. Cells were cultivated in McCoy's 5a medium supplemented with 15% FCS, 1% l-glutamine and 1% non-essential amino acids (all from Biochrome, Germany) and without antibiotics in uncoated 12 well-plates, and were allowed to rest overnight prior to experiments (37°C, 7% CO2). Cells were infected with a 5 × MOI of stationary phase *L. major*. Because of the reported lower affinity of the human AhR compared to murine AhR (29), we added 10 μl of 30 mM solutions of AhR ligands or carrier per ml of medium.

### *Real-Time* PCR and Microarray Analysis

RNA from GMΦ and human monocytes was extracted using the Quiagen RNeasy micro kit according to the manufacturer's instructions. For RNA extraction from mouse feet, skin was homogenated in RNeasy lysis buffer using a peqlab Precellys homogenizer for a single run at maximum power and time settings. Semi- quantitative RT-PCR was performed as described previously on a Bio-Rad CFX384 Touch Real-Time PCR detection system (28).

For microarray analysis, total RNA from three independent experiments of 4 h *L. major* infected C57BL/6 or BALB/c GMΦ was isolated and subsequently processed for microarray hybridization using Affymetrix Murine Genome MG_U74Av2 arrays according to the manufacturer's instructions (Affymetrix). Arrays were developed and analyzed as previously described (5). Microarray data were analyzed using MicroArray Suite Software 5.0 (Affymetrix) using data from corresponding control samples as baseline.

We retained only genes which were significantly regulated in every single experiment (change *p*-value < 0.05, fold-change ≥1.5, expression over background) as well as in the complete set of experiments (fold-change of ≥1.5, *p*-value of < 0.05, paired *t*-test).

To compare *L. major* induced alterations expression patterns between macrophages isolated from resistant and susceptible mouse strains, signal log ratios of infected vs. uninfected control samples in both mice strains were evaluate by paired *t*-test. We retained only genes with a *p* < 0.05 and a differential fold-change regulation of ≥1.5.

To identify transcription factors with statistically over-represented binding sites in promoter regions of regulated genes, we used CARRIE with the implemented promoter sequence analysis tool ROVER (30). Promoter sequences were defined 1000 bases upstream to 100 bases downstream of the transcription start site and obtained using PromoSer (31) and equally sized group of control genes with stable expression but no detectable regulation by *L. major* infection were compared.

## Results

### Transcriptional Changes in *L. major* Infected GMΦ

To identify genes that are regulated during infection of macrophages with *L. major*, GMΦ from C57BL/6 and BALB/c mice where incubated with metacyclic *L. major* for 4 h with a multiplicity of infection (MOI) of 5:1. More than 75% of macrophages had taken up *L. major* parasites at this timepoint. Using a cut-off value of 1.5-fold, 141 genes were significantly upregulated and 127 were significantly downregulated in GMΦ from C57BL/6 mice, whereas 69 genes were upregulated and 91 where downregulated in GMΦ from BALB/c mice. Among upregulated genes we found *Tnf* and other cytokine genes, transcription factors, genes associated with apoptosis, lipid metabolism and the NF-kappa B cascade. Table 1 shows a selection of upregulated genes. Array data was uploaded to the NCBI GEO repository under accession number GSE127541. Supplemental Tables 1, 2 contain a complete list of regulated genes in C57BL/6 and BALB/c mice, respectively.

**Table 1:** Genes significantly upregulated by *Leishmania major* infection.

**Gene Symbol**	**Description (NCBI gene)**	***n*-fold C57BL/6**	***n*-fold BALB/c**	***p*-value for differential regulation in C57BL/6 vs. BALB/C**
**Chemokines and receptors**				
Ccl4	Chemokine (C-C motif) ligand 4	24.9	8.7	n.s
Cxcl2	Chemokine (C-X-C motif) ligand 2	21.0	12.0	n.s.
Cxcl1	Chemokine (C-X-C motif) ligand 1	17.9	10.7	n.s.
Ccrl2	Chemokine (C-C motif) receptor-like 2	2.6	3.2	n.s.
**Cytokines and related molecules**				
Tnf	Tumor necrosis factor	18.2	8.2	n.s.
Il1a	Interleukin 1 alpha	10.7	6.1	n.s.
Il1rn	Interleukin 1 receptor antagonist	5.1	3.2	n.s.
**Apoptosis**				
Myc	Myelocytomatosis oncogene	2.7	−0.2	0.007
Tnfaip3	Tumor necrosis factor, alpha-induced protein 3	4.3	2.8	n.s.
Socs3	Suppressor of cytokine signaling 3	3.9	2.3	n.s
Casp4	Caspase 4, apoptosis-related cysteine protease	2.3	1.7	n.s.
Sod2	Superoxide dismutase 2, mitochondrial	2.3	1.6	n.s.
Cdkn1a	Cyclin-dependent kinase inhibitor 1A (P21)	1.9	1.6	n.s.
**Receptors and cell surface proteins**				
Vcam1	Vascular cell adhesion molecule 1	10.6	3.0	n.s.
Olr1	Oxidized low density lipoprotein (lectin-like) receptor 1	9.0	2.5	<0.001
Icam1	Intercellular adhesion molecule	3.3	1.7	n.s.
Adora2a	Adenosine A2a receptor	3.2	2.2	n.s.
**Mapkkk cascade**				
Gadd45b	Growth arrest and DNA-damage-inducible 45 beta	3.1	2.8	n.s.
Cav	Caveolin, caveolae protein	2.3	0.7	0.008
Mapkapk2	MAP kinase-activated protein kinase 2	2.0	1.5	n.s.
Dusp1	Dual specificity phosphatase 1	2.1	1.5	n.s.
**Other genes involved in immune response**				
Tnip1	TNFAIP3 interacting protein 1	2.5	1.7	n.s.
Traf5	Tnf receptor-associated factor 5	2.3	1.6	n.s.
Slfn2	Schlafen 2	3.1	0.7	0.034
Mmp13	Matrix metalloproteinase 13	2.5	1.6	0.031
Ifi205	Interferon activated gene 205	2.2	−1.1	0.001
Traf1	Tnf receptor-associated factor 1	12.8	8.1	0.049
**Lipid metabolism**				
Ptgs2	Prostaglandin-endoperoxide synthase 2, cox-2	47.2	8.8	n.s.
Ptges	Prostaglandin E synthase	3.8	3.0	n.s.
**Transcription factors**				
Fosl1	Fos-like antigen 1	14.9	4.2	n.s.
Ahr	Aryl-hydrocarbon receptor	8.7	4.6	0.020
Spic	Spi-C transcription factor (Spi-1/PU.1 related)	7.4	4.4	n.s.
**Protein import into nucleus/nf-kappa b cascade**				
Nfkbia	Nuclear factor of kappa light chain gene enhancer in B-cells inhibitor, alpha	3.8	2.7	n.s.
Nfkbib	Nuclear factor of kappa light chain gene enhancer in B-cells inhibitor, beta	2.2	1.7	n.s.
Kpna3	Karyopherin (importin) alpha 3	2.0	1.8	n.s
Nfkb2	Nuclear factor of kappa light polypeptide gene enhancer in B-cells 2, p49/p100	2.3	1.8	n.s
Nfkb1	Nuclear factor of kappa light chain gene enhancer in B-cells 1, p105	2.3	1.8	n.s

### Infected GMΦ From Resistant Mice Upregulate *Ahr*

While most genes were similarly regulated in resistant and susceptible mice, we found only eight genes that showed strain-specific differences. Among them was the transcription factor *Ahr*.

To check whether AhR-signaling could be involved in the underlying transcriptional regulatory networks, we analyzed overrepresentation of transcription factor binding sites among regulated genes using CARRIE analysis, and found AhR binding sites overrepresented in upregulated C57BL/6 genes with a *p*-value of 0.000372 (Table 2).

**Table 2:** Top ten overrepresented transcription factor binding sites among genes upregulated in *Leishmania major*-infected C57BL/6 granuloma macrophages.

**Binding site**	***p*-value**
NF-kappaB binding site	3.14E-08
Myogenic enhancer factor 2	0.00000382
Cellular and viral TATA box elements	0.00000391
Signal transducer and activator of transcription 1	0.00000634
Paired box factor 2	0.00000805
c-Rel	0.000148
ATF-1	0.000159
AhR	0.000372
BTB and CNC homolog 1	0.000526
HNF-3/Fkh homolog-8	0.000621

Therefore, and because AhR has recently been identified as an important regulator of innate immunity (22, 26, 32–34) and was implicated in regulation of resistance to experimental leishmaniasis (24, 35, 36), we focused on AhR.

**Figure 1:**
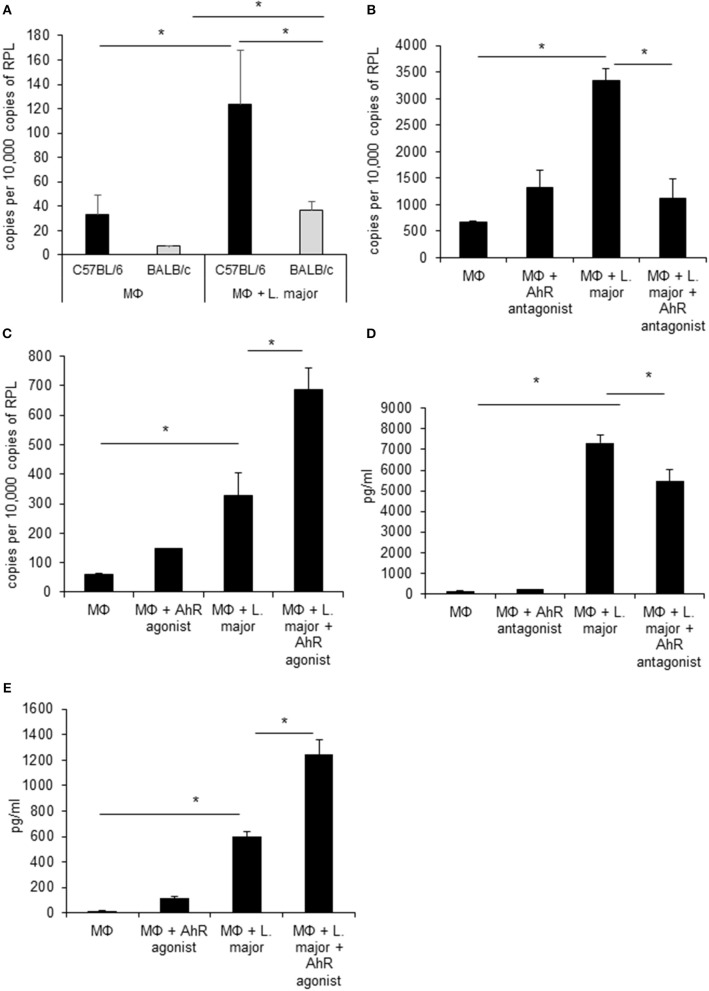
Infected granuloma macrophages express AhR upon infection with *L. major*, which regulates *Tnf* α expression and secretion. RT-PCR data showing differential induction of AhR in granuloma macrophages from C57BL/6 (black bars) and BALB/c mice (grey bars) infected with a 5 × MOI of metacyclic *L. major* for 24h **(A)**. RT-PCR of *Tnf* mRNA expression from C57BL/6 macrophages treated with AhR antagonist CH-223191 **(B)** and AhR agonist ITE **(C)** after incubation with a 5 × MOI of metacyclic *L. major* for 4 h **(B)**. TNF secretion by macrophages from C57BL/6 mice infected with *L. major* for 24 h in the presence of 30 nmol/ml AhR antagonist CH-223191 **(D)** and TNF secretion by macrophages infected with *L. major* for 24 h in the presence of 30 nmol/ml AhR agonist ITE **(E)**. ^*^p < 0.05, *n* = 3. Data shown is representative of three independent experiments with similar results.

We first confirmed gene regulation of *Ahr* in GMΦ using *RT-PCR*. In agreement with microarray data, uptake of *L. major* induced *Ahr* expression significantly in both mice strains. *Ahr* induction was significantly stronger in C57BL/6 vs. BALB/c mice (Figure 1A). The strain specific difference in AhR upregulation between GMΦ from resistant and susceptible mice indicated a possible beneficial role of AhR in resistance to *Leishmania*.

### AhR Ligands Change Cytokine Secretion by *L. major* Infected Macrophages

**Figure 2:**
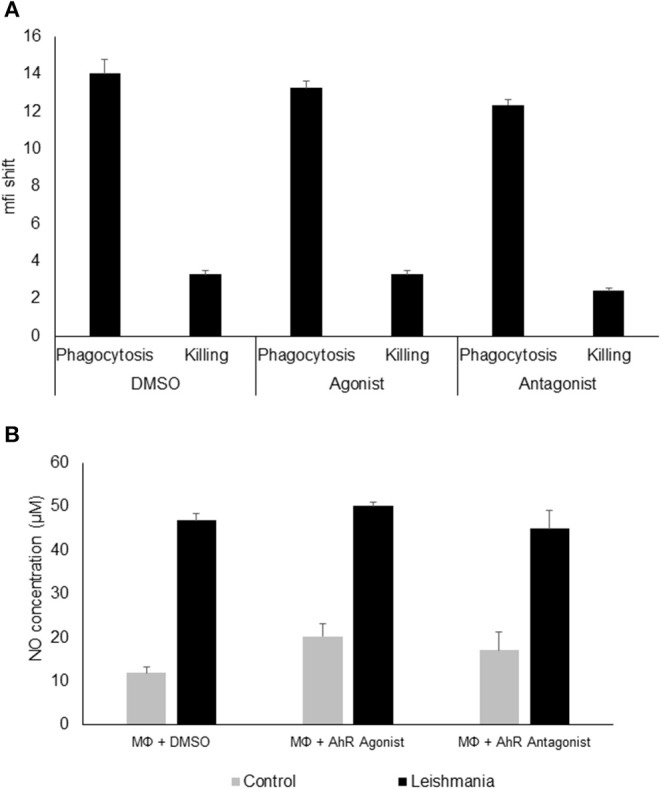
AhR ligands do not affect phagocytosis, killing or NO production in granuloma macrophages. FACS analysis of granuloma macrophages incubated with a 5 × MOI of FITC-stained metacyclic *L. major* parasites in the presence of DMSO (carrier control), AhR agonist ITE or AhR antagonist CH-223191 for 4 h (phagocytosis) and after washing and further 20 h incubation in the presence of 500 units rmIFNγ (killing). Graphs display the mean fluorescence intensity shift in the FITC channel gated on macrophages incubated with stained parasites as compared to uninfected controls **(A)**. Measurement of NO production of macrophages prestimulated with 500U of rmIFNγ for 1 d followed by infection with 5 × MOI of *L. major* for 24 h treated with AhR agonist ITE, AhR antagonist CH-223191 and carrier control (DMSO) in the presence of 500U of rmIFNγ **(B)**, *n* = 3.

Since AhR itself presents a transcription factor and AhR binding sites were overrepresented among *L. major*-induced genes, we checked for relevance of AhR in *Leishmania*-induced gene expression. To this end we analyzed gene expression of C57BL/6 derived GMΦ after treatment with AhR antagonist CH-223191. We observed a reduction in *Tnf* expression (Figure 1B). Conversely, treatment with the AhR agonist (2-(1*H*-Indol-3-ylcarbonyl)-4-thiazolecarboxylic acid methyl ester (ITE) caused a higher expression of *Tnf* in macrophages co-incubated with *L. major* for 4 h (Figure 1C). Subsequently, we analyzed the effects of AhR antagonist CH-223191 (Figure 1D) or AhR agonist ITE (Figure 1E) on production and release of TNF protein by infected GMΦ. As shown previously using intracellular FACS analysis in GMΦ (8), GMΦ secreted TNF protein upon *L. major* infection. As indicated by RT-PCR data we found reduced secretion of TNF protein by cells treated with AhR antagonist, while those treated with AhR agonist showed enhanced secretion. In terms of general macrophage function, FACS analysis with FITC-stained parasites indicates that most cells contain parasites after 4 h, regardless of AhR ligand treatment. Macrophages are usually not able to effectively eliminate parasites since *L. major* inhibits upregulation of inducible nitic oxide synthase necessary for parasite killing (37). Also, since it has been observed that *Ahr* knockout peritoneal macrophages produce less NO upon LPS/IFNγ stimulation (26), we analyzed parasite killing and NO production in *L. major*/IFNγ stimulated cells. However, incubation for 20 h in the presence of IFNγ revealed no obvious differences in killing between cells treated with agonist or antagonist and untreated cells (Figure 2A; Supplemental Figure 1). Similarly, *Leishmania*-induced NO production in IFNγ-stimulated cells was unaffected by treatment with either agonist or antagonist (Figure 2B).

### *L. major* Infected Human Blood Monocytes Do Not Upregulate *Ahr* but AhR Antagonist Treatment Reduces *Tnf* Expression

**Figure 3:**
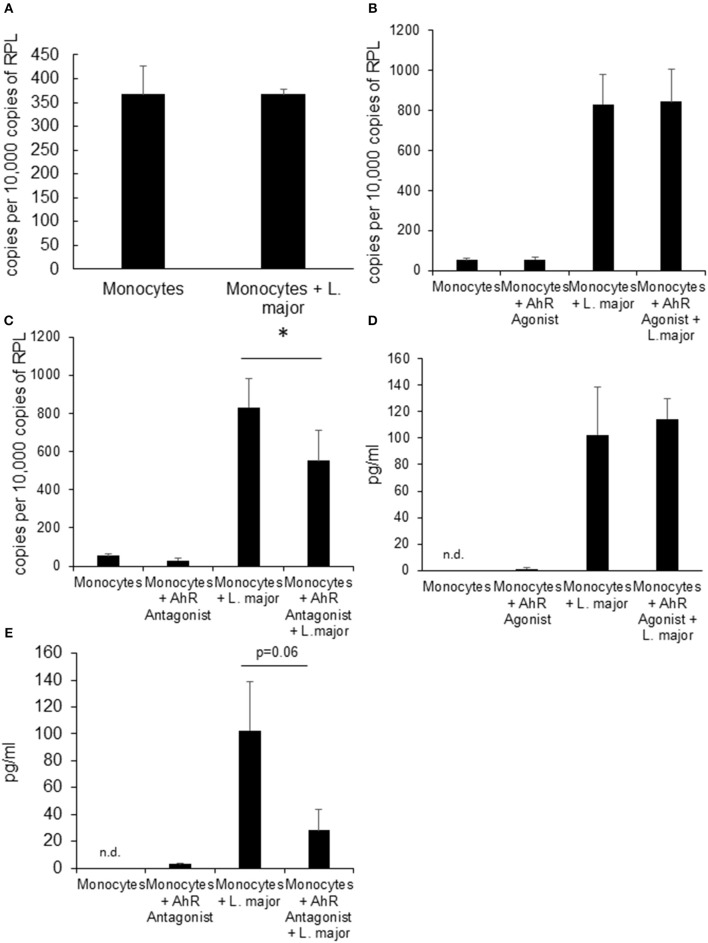
Human monocytes do not upregulate *Ahr* upon infection with *L. major* but treatment with AhR antagonist reduces *Tnf* expression. RT-PCR data showing no induction of *Ahr* in human blood monocytes infected with a 5 × MOI of metacyclic *L. major* for 24 h **(A)**. RT PCR of *Tnf* mRNA expression from human monocytes treated with AhR agonist ITE **(B)** and antagonist CH223191 **(C)** after incubation with 5 × MOI of metacyclic *L. major* for 4 h. TNF concentration in the supernatant of human blood monocytes infected with a 5 × MOI after 4 h of infection in the presence of AhR agonist ITE **(E)** and antagonist CH223191 **(D)**. The graphs represent cumulative data from 3 independent experiments from individual blood donors. ^*^p < 0.05.

We also treated human monocytes co-incubated with *Leishmania* parasites with AhR agonist and antagonist in order to assess the translational potential of our findings in murine granuloma macrophages. We could not detect upregulation of *Ahr* in human monocytes from healthy donors in response to *in vitro Leishmania* infection (Figure 3A). While *Tnf* expression was induced in infected monocytes after 4 h, there was no additional effect of treatment with AhR agonist ITE (Figure 3B). However, we could observe a reduced induction of *Tnf* expression in human monocytes treated with AhR antagonist CH-223191 (Figure 3C). When measuring TNF secretion after 24 h using ELISA, variability was very high between samples and there were no differences reaching statistical significance. As the kinetics might be different between these cell types, we measured TNF secretion after 4 h and found no difference with agonist treatment (Figure 3D). When treated with AhR antagonist however, there was a trend toward reduced TNF secretion (Figure 3E), but it failed to reach our significance threshold with *p* = 0.06.

### Treatment of Infected Mice With AhR Ligands Influences the Cytokine Milieu *in vivo*

**Figure 4:**
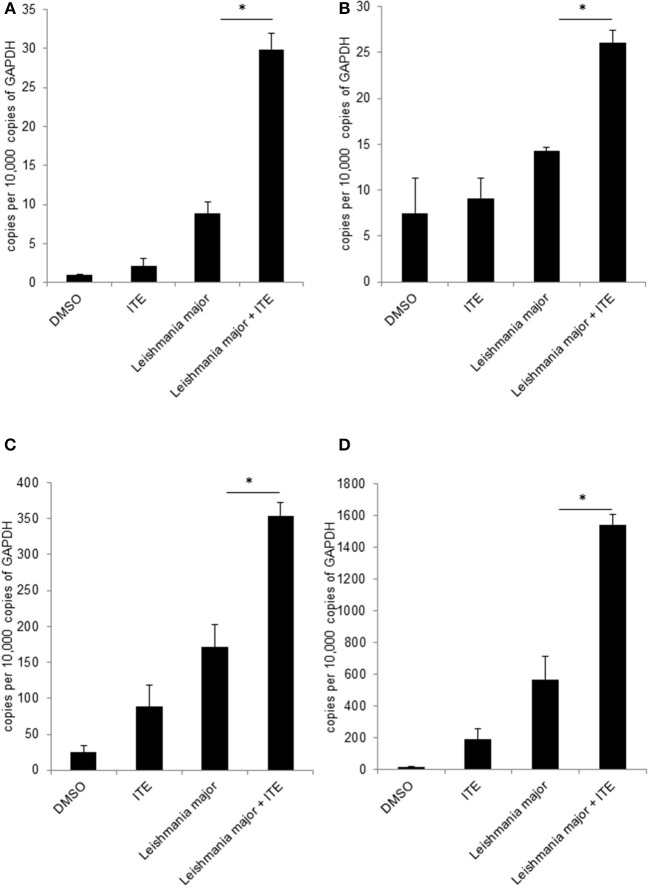
Early local AhR treatment changes the local cytokine milieu. Susceptible BALB/c mice were treated with 1 μl of 30 mM (30 nmol) solution of AhR agonist ITE during inoculation with 2 × 10^7^ *L. major*. Skin samples were taken after 20 h of infection for RT-PCR analysis. We show expression levels of *Tnf* **(A)**, *Cox2* **(B)**, *Cxcl2* **(C)** and *Cxcl10* **(D)**. ^*^p < 0.05, *n* = 3. Data shown is representative of three independent experiments with similar results.

Having shown that *Ahr* is involved in *L. major*-induced gene expression by macrophages and that it increases release of TNF, we addressed our concept that gene expression in macrophages is decisively involved in generating a resistance-inducing early cutaneous micromilieu *in vivo*. Since we had previously demonstrated that *L. major* infection resulted in an induction of cytokines in infected skin (5), we analyzed whether interference with AhR-signaling modulates induction of these cytokines. Therefore, we injected BALB/c mice with AhR agonist ITE during infection with *L. major* and measured expression of genes using *RT-PCR* of foot skin samples after 20 h. We observed a significant increase in expression of *Tnf, Cox2 Cxcl2* and *Cxcl10* (Figures 4A–D). We demonstrated AhR-dependent induction of both well-established (*Cox2*), but also novel AhR target genes (*Cxcl10*) in *L. major*-infected skin, while expression of other genes (*Il12, Il10, Il4, Il6, Il1*β*, Ccxcl11, Il1a*, and *Cxcl1*) was not influenced (data not shown). Thus, signaling by AhR selectively induces genes which are known to be markedly present in the early cutaneous micromilieu after *L. major* infection.

### Treatment of Infected Mice With AhR Ligands Increases Resistance to *L. major* Infection in BALB/c Mice

**Figure 5:**
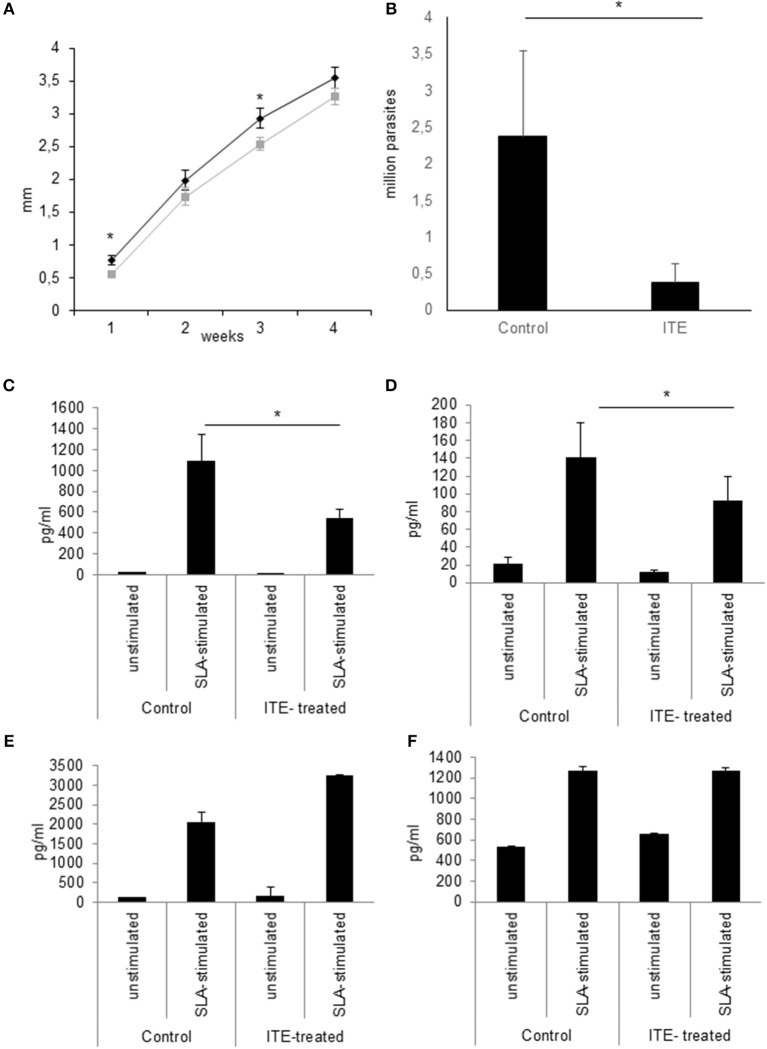
Early local AhR treatment reduces infection severity in susceptible animals Treatment of susceptible BALB/c mice (*n* = 5 per group) with 30 nmol of AhR agonist ITE during inoculation with 2 × 10^7^ *L. major*. Course of the infection was monitored by measuring the foot swelling in mm **(A)**. Differences in parasite dissemination were measured by limiting dilution assay of popliteal lymph node cells after 4w, *n* = 5 **(B)**. The quality of the T-cell response was assessed by measuring the secretion of Th2 cytokine IL4 by popliteal draining lymph node cells restimulated with soluble *Leishmania* antigen for 5d after 11d **(C)** and at w4 experiments **(D)**. Th1 cytokine IFNγ was measured accordingly **(E,F)**. ^*^p < 0.05, *n* = 5. Data shown is representative of 5 independent experiments with similar results.

**Figure 6:**
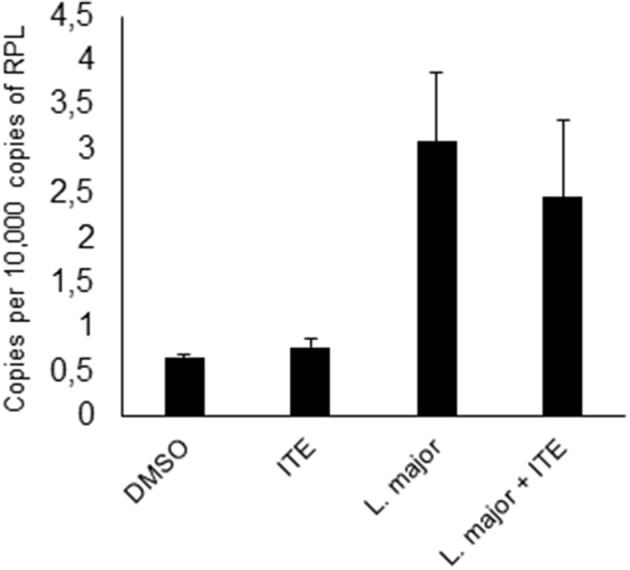
Early local AhR agonist- treatment induced differences in *Tnf* expression are transient. Susceptible BALB/c mice were treated with 1 μl of 30 mM (30 nmol) solution of AhR agonist ITE during inoculation with 2 × 10^7^ *Leishmania major*. Skin samples were taken after 3w of infection for RT-PCR analysis. *n* = 5.

Since increased *L. major*-induced gene-expression in C57BL/6 mice in comparison to BALB/c mice is associated with resistance (5) and AhR-induced TNF is one known decisive agent in leishmaniasis (38–41), we wondered if susceptible mice could benefit from local AhR activation during early leishmaniasis. Therefore, we treated susceptible BALB/c mice with AhR agonist ITE, given as a single injection at the time of infection, reflecting its rapid induction in GMΦ of resistant mice. We observed a rapid increase in footpad swelling typical for high dose infection with this *L. major* strain. Importantly, we observed a small, but significant reduction in foot swelling during the first weeks of infection (Figure 5A), and, more relevantly, also more than 4-fold reduced parasite loads in dLN after 4w of infection (Figure 5B). We did not detect differences in parasite loads earlier during infection (11d, not shown). However, we were able to demonstrate reduced secretion of Th2 cytokine IL4 by popliteal lymph node cells restimulated with soluble *Leishmania* antigen (SLA), both early after parasite inoculation (11d, Figure 5C) and in established infection (4w, Figure 5D). Secretion of Th1 cytokine IFNγ was increased early after parasite inoculation, but this difference did not reach statistical significance and could not be observed in established infection (Figures 5E,F). This indicates that the Th2 response in AhR agonist-treated BALB/c mice is diminished, albeit not completely switched toward a Th1 response. In agreement with this, both the differences in foot swelling and parasite loads diminished during prolonged infection after 4 weeks (data not shown). To ascertain the transient nature of AhR agonist single treatment- induced differences in lesional *Tnf* expression, we also measured *Tnf* expression in the skin after 3w of infection. While there is residual *Tnf* expression in the lesion at this time point, we found no difference between treated and untreated animals (Figure 6).

## Discussion

In order to identify how macrophages contribute to the early cutaneous cytokine milieu, we analyzed *L. major*-induced gene expression profiles of tissue macrophages from resistant and susceptible mice. Using this experimental approach, we demonstrated that *L. major* infection caused a marked induction of several immune modulatory genes in tissue macrophages from both mice strains. Different macrophage subtypes respond differently to *L. major* and so far, *L. major*-induced gene expression profiles were analyzed using bone marrow-derived or peritoneal macrophages. In contrast to data from bone marrow macrophages infected for 24 h (13), we did not detect a general suppression of gene expression, but the number of repressed and induced genes was rather similar after 4 h of infection. This supports the observation that a dominant suppression of macrophage gene expression occurs only during prolonged infection (13).

Upregulation of several inflammatory cytokines has been seen as part of a global gene expression response in bone marrow-derived macrophages from resistant and susceptible strains infected with a Tunisian isolate of *L. major* (42). Their *L. major*-induced gene expression profile was otherwise distinct from our results. These differences probably reflect the heterogeneity of different macrophage and parasite populations and stress the importance of clarifying the physiological significance of *in vitro* data using *in vivo* models.

In our *L. major*-induced gene expression profile we found that most genes were similarly regulated in resistant and susceptible mice (Table 1). Only eight genes showed strain-specific differences, among them the transcription factor *Ahr*, which was more strongly upregulated in macrophages from resistant animals. AhR is emerging as an important regulator in host defense and homeostasis [for a review, see (43)]. It was also recently described as a regulator in macrophage polarization (26) and several publications have reported a role for AhR in resistance to experimental Leishmaniasis (24, 35, 36).

Since *Ahr* expression was induced, and since we also found a significant overrepresentation of AhR binding sites among promoter regions of genes upregulated by *L. major* infection, we expected that AhR could be involved in regulating *L. major*-induced gene expression in macrophages. Both positive and negative interaction with important inflammatory transcription factors like the Nf-kappa-B family in myeloid cells have been described for AhR (44). AhR activity has been shown to attenuate macrophage cytokine production in response to LPS (25, 32, 34). Similarly, peritoneal macrophages from *Ahr* knockout mice produced higher amounts of TNF and other cytokines in response to IFNγ and LPS and showed lower expression of M2 markers upon IL4 stimulation (26). Importantly, while TNF is induced in LPS-stimulated *Ahr* knockout macrophages, production of NO is inhibited, indicating an ambiguous nature of AhR-signaling in terms of macrophage functions relevant for Leishmaniasis. There are also reports that AhR ligand TCDD induces TNF production in the human macrophage cell line THP1 via the AhR pathway (45), underlining the complexity of the relationship of AhR activity with TNF production.

We now demonstrate that activation of AhR using non-persistent pharmacological AhR agonist ITE enhances TNF production in GMΦ, while treatment with AhR antagonist CH-223191 diminishes TNF production during *L. major* infection. This is seemingly contradictory to the upregulation of TNF observed in IFNγ/LPS- stimulated macrophages from *Ahr* knockout mice, which would suggest a suppressive role of AhR in macrophage TNF secretion (26), but as mentioned above, TCDD induces TNF in human THP1 cells via activation of AhR (45), so whether AhR activity induces or suppresses TNF might well-depend on the specific macrophage type, activation status and stimulus. The observation that *Ahr* knockout peritoneal macrophages produce less NO upon LPS/IFNγ stimulation (26) indicates that AhR activity enhances NO production in macrophages. However, in our experiments, IFNγ- activated granuloma macrophages treated with AhR ligands did not exhibit changes in NO secretion upon infection with *L. maj*or. The lack of difference in NO production was accompanied by a lack of difference in parasite load of infected, IFNγ-activated macrophages treated with either ligand. These differences in macrophage responses might again reflect the heterogeneity of macrophage subtypes and response patterns to different stimuli (46).

We also tested the role of AhR-signaling in human monocytes. *Ahr* mRNA was not upregulated in human blood monocytes upon infection but AhR antagonist significantly reduced expression of *TNF* mRNA while agonist treatment had no effect. Thus, the effects were not analogous to the response of murine granuloma macrophages but the reduction of *TNF*-mRNA nevertheless indicates a possible role of AhR-signaling in human *L. major* infected monocytes. These data are not yet sufficient to support the concept that AhR-signaling has pathophysiological effects in human leishmaniasis. Extensive studies using different human monocyte/macrophage populations and parasite strains are needed to answer this question.

Thus, as *in vitro* data on AhR-signaling suggest both positive and negative effects on macrophage activation, and our own data suggested a positive regulation of *Leishmania*-induced cytokine secretion by AhR, we then investigated the relevance of AhR-signaling during *L. major* infection *in vivo*. Analyzing the effect of AhR activation on cytokine expression in infected skin, we found that AhR agonist indeed also induced expression of *Tnf in vivo*. We also observed AhR-dependent regulation of *Cxcl10* and *Cxcl2*. We have previously demonstrated that *Tnf*, *Cxcl10* and *Cxcl2* are all more prominently expressed in the skin of resistant animals (5) and could therefore contribute to resistance. Other, already confirmed early regulatory cytokines like Il4, Il6, Il12, and Il1a (3, 5, 47) were not affected by ITE treatment.

Thus, AhR activity contributes to the early skin cytokine milieu. Since we previously had demonstrated the relevance of the early cutaneous cytokine milieu for the subsequent development of resistance (5, 6), we speculated that early AhR-dependent signaling also influences the course of infection. Indeed, when we treated susceptible BALB/c mice with AhR agonist ITE at the first day of infection we observed a small, but significant reduction in footpad swelling during the first and third week of infection. No more differences in footpad swelling were observed after 4w, but we found more than 4-fold reduced numbers of parasites in skin dLN. We also found a significant~50% reduction of *L. major*-specific IL4 secretion by restimulated dLN-cells in the early phase of Th differentiation. The relative reduction in IL4 secretion by ITE treatment was still observable after 4w, albeit not as pronounced. We observed no concomitant increase of IFNγ secretion. The reduced levels of IL-4 could enhance macrophage effector functions and explain the lower numbers of living parasites in draining lymph nodes. Thus, we show significant and matching effects on footpad swelling, parasite levels and the Th2 response during the first weeks of infection after a very short and local treatment with non-persistent AhR agonist only at the time of parasite inoculation. Therefore, our experiments indicate that AhR activation in skin of susceptible mice enhances early resistance against *L. major* by diminishing the Th2 response, but is not sufficient to reverse the genetic susceptibility toward the parasite.

Significant involvement of AhR-signaling in immunity (23, 33, 48) is established for infection with e.g., influenza and toxoplasma. There are also some data on the relevance of AhR for experimental leishmaniasis. *Ahr* knockout mice on a resistant background showed increased resistance compared to wildtype animals (24). On the other hand, oral treatment of infected mice with persistent AhR ligand 2,3,7,8-Tetrachlorodibenzo-p-dioxin (TCDD) lead to a slower disease progression and lower parasite numbers not only in BALB mice, but also in Skid mice lacking T-cells, indicating that TCDD-induced changes in AhR activity in innate immune cells are relevant for AhR-mediated resistance (35).

While these studies demonstrate that AhR-signaling can influence experimental leishmaniasis, seemingly both positively and negatively, and in case of peritoneal knockout macrophages, at the same time, they focus neither on the function of AhR-signaling during the early crucial phase of infection nor on the relevance of AhR-signaling at the site of infection. The work by Elizondo et al. using *Ahr* knockout mice on a resistant background found that systemic absence of AhR caused a decrease in FoxP3+ regulatory T-cells in infected animals in the 4th and 6th week of infection and a concomitant increase in IL10 secretion by restimulated pLN cells after 6w, while Leishmania Ag specific secretion of IFNγ and IL4 were not affected in infected animals (24). Moreover, *Ahr* knockout mice had higher serum levels of TNF in the blood prior to and also during infection. The source of TNF production was not specifically determined. Since in our experiments AhR was only activated locally in the early phase of infection prior to the development of the Th-cell response, these results are not necessarily contradictory to our observed beneficial effect of early local AhR activation, especially because there is evidence for AhR activity-dependent induction of TNF secretion in macrophage like cells (45). The permanent absence of AhR-signaling in all immune cells including T-cells does not allow for analyzing time- or location-dependent effects. In those studies using animals treated systemically with permanent AhR ligand TCDD, AhR is permanently activated. Since effects of TCDD were also seen in SCID mice, this argues for T-cell independent effects of AhR-signaling in experimental leishmaniasis. However, significant differences in parasite numbers were only observed after 4 weeks using high doses of TCDD in a low dose infection model and may therefore reflect the prolonged activation of AhR in this model which is in contrast to the transient activation by ITE in our experiments. Also, there is recent evidence from *Ahr* knockout rats suggesting that the classical AhR ligand TCDD has AhR independent effects on a number of immune cells like CD11^+^ cells (49). Thus, the results from experiments using the TCDD are not in contrast to our results indicating an effect of early local AhR-signaling on Th-cell differentiation.

Our data newly establish that the local signaling by AhR increases resistance early during experimental leishmaniasis. It may exert these effects by influencing mechanisms of both innate and adaptive immunity. It has long been known, that early TNF production is required for resistance (38–41). Thus, enhanced expression of TNF by infected GMΦ might contribute to the increased early resistance in our *in vivo* experiments. TNF is necessary to facilitate the leishmanicidal properties of macrophages by inhibiting the IL4-induced production of Arg1 (50). Here, the lesional macrophage phenotype of TNF-deficient animals closely resembled that of susceptible BALB/c mice, so AhR activation might ameliorate the low leishmanicidal phenotype by boosting local TNF secretion. However, this effect can only be responsible for the very early difference in footpad swelling, as ITE is not a persistent AhR ligand and we have shown that TNF levels do not remain higher in ITE-treated animals during the height of infection.

With respect to adaptive immunity, we used ITE, a non-persistent AhR agonist which does not lead to TCDD-induced lymphocyte suppression and toxicity. We demonstrate significantly reduced *L. major*-specific secretion of IL4 by dLN-cells and therefore a diminished early Th2-response. We already demonstrated that differences in the early skin micromilieu correlate with differences in cytokine secretion from dendritic cells in skin draining lymph nodes (5). Thus, a similar effect of AhR-signaling on adaptive immunity via the altered skin cytokine milieu and dendritic cells is possible. The exact mechanisms of AhR-dependent effects on adaptive immunity have to be revealed in further studies.

In summary, our experiments demonstrate the relevance of AhR-signaling for cytokine expression in murine macrophages. We also demonstrate that AhR antagonist treatment reduced *L. major* induced *TNF* mRNA expression in human monocytes, indicating a possible role of AhR-signaling in the response of human monocytes to this parasite. *In vivo* we demonstrate the relevance of AhR-signaling in skin during *L. major* infection and provide further evidence for a relevance of the early skin micromilieu in instructing resistance to *L. major*.

Importantly, we showed that local treatment with a non-toxic, non-persistent, physiological AhR ligand had beneficial effects on experimental leishmaniasis in susceptible mice, opening possibilities of further studies on the effects of AhR-signaling in infectious diseases like leishmaniasis.

## Data Availability Statement

The array data generated for this manuscript was uploaded to the NCBI GEO repository under accession number GSE127541: https://www.ncbi.nlm.nih.gov/geo/query/acc.cgi?acc=GSE127541.

## Ethics Statement

Human monocytes were isolated from fresh human blood leukocyte reduction chambers of platelet apheresis sets from healthy, voluntary whole blood donations after informed consent of the donors according to the regulations of the blood bank of the University Hospital Münster. Leukocyte reduction filters were anonymized prior to delivery from the blood bank in line with the ethics code provided by the scientific and ethics committee of the University of Münster.

## Author Contributions

N-AM, JR, CS, and JE contributed to the design and conception of the study. JE acquired and analysed array data. N-AM acquired and analysed all other data. N-AM wrote the first draft of the manuscript. N-AM, JE, and CS wrote sections of the manuscript. N-AM, JR, CS, and JE contributed to manuscript revision, read, and approved the submitted version.

### Conflict of Interest

The authors declare that the research was conducted in the absence of any commercial or financial relationships that could be construed as a potential conflict of interest.
