# Supplementary material for: Aryl Hydrocarbon Receptor-Signaling Regulates Early Leishmania major-Induced Cytokine Expression
Source: Front Immunol. 2019 Oct 15;10:2442. doi: 10.3389/fimmu.2019.02442 (PMC6843081; doi:10.3389/fimmu.2019.02442)
Supplement: Supplementary file 1 [file Table_1.docx]

Supplementary Material

# Supplementary Table 1

| Supplementary Table 1: Genes regulated in 4h *Leishmania major* infected granuloma macrophages from C57BL/6 mice | | |
| --- | --- | --- |
| Gene name | Description | n-fold |
| Ptgs2 | prostaglandin-endoperoxide synthase 2 | 47.17 |
| Ccl4 | chemokine (C-C motif) ligand 4 | 24.85 |
| Cxcl2 | chemokine (C-X-C motif) ligand 2 | 20.99 |
| Tnf | tumor necrosis factor | 18.17 |
| Cxcl1 | chemokine (C-X-C motif) ligand 1 | 17.87 |
| Cxcl1 | chemokine (C-X-C motif) ligand 1 | 16.78 |
| Fosl1 | fos-like antigen 1 | 14.93 |
| Irg1 | immunoresponsive gene 1 | 14.07 |
| Traf1 | Tnf receptor-associated factor 1 | 12.77 |
| Il1a | interleukin 1 alpha | 10.68 |
| Vcam1 | vascular cell adhesion molecule 1 | 10.62 |
| Phlda1 | pleckstrin homology-like domain. family A. member 1 | 9.08 |
| Olr1 | oxidized low density lipoprotein (lectin-like) receptor 1 | 9.02 |
| Ahr | aryl-hydrocarbon receptor | 8.66 |
| Slc7a11 | solute carrier family 7 (cationic amino acid transporter. y+ system). member 11 | 7.61 |
| Spic | Spi-C transcription factor (Spi-1/PU.1 related) | 7.36 |
| Irg1 | immunoresponsive gene 1 | 7.16 |
| Serpinb2 | serine (or cysteine) proteinase inhibitor. clade B. member 2 | 6.25 |
| Clecsf9 | C-type (calcium dependent. carbohydrate recognition domain) lectin. superfamily member 9 | 6.25 |
| Ehd1 | EH-domain containing 1 | 5.54 |
| Il1rn | interleukin 1 receptor antagonist | 5.12 |
| Mlp | MARCKS-like protein | 4.85 |
| Ets2 | E26 avian leukemia oncogene 2. 3' domain | 4.43 |
| Npn3 | neoplastic progression 3 | 4.43 |
| Tnfaip3 | tumor necrosis factor. alpha-induced protein 3 | 4.27 |
| Snk | serum-inducible kinase | 4.13 |
| Pcdh7 | protocadherin 7 | 4.04 |
| Ralgds | ral guanine nucleotide dissociation stimulator | 3.91 |
| Socs3 | suppressor of cytokine signaling 3 | 3.91 |
| Nfkbia | nuclear factor of kappa light chain gene enhancer in B-cells inhibitor. alpha | 3.81 |
| Ptges | prostaglandin E synthase | 3.79 |
| Nfkbia | nuclear factor of kappa light chain gene enhancer in B-cells inhibitor. alpha | 3.60 |
| Ugcg | UDP-glucose ceramide glucosyltransferase | 3.49 |
| Mail-pending | molecule possessing ankyrin-repeats induced by lipopolysaccharide | 3.33 |
| Icam1 | intercellular adhesion molecule | 3.31 |
| Adora2a | adenosine A2a receptor | 3.21 |
| Mt2 | metallothionein 2 | 3.18 |
| Ifrd1 | interferon-related developmental regulator 1 | 3.11 |
| Slfn2 | schlafen 2 | 3.09 |
| Gadd45b | growth arrest and DNA-damage-inducible 45 beta | 3.08 |
| Slc11a2 | solute carrier family 11 (proton-coupled divalent metal ion transporters). member 2 | 3.03 |
| Ak4 | adenylate kinase 4 | 3.01 |
| Ampd3 | AMP deaminase 3 | 2.98 |
| Slfn2 | schlafen 2 | 2.90 |
| Bdnf | brain derived neurotrophic factor | 2.90 |
| Cd83 | CD83 antigen | 2.85 |
| Pcsk5 | proprotein convertase subtilisin/kexin type 5 | 2.84 |
| Myc | myelocytomatosis oncogene | 2.72 |
| Fabp3 | fatty acid binding protein 3. muscle and heart | 2.68 |
| Mcoln2 | mucolipin 2 | 2.64 |
| Ccrl2 | chemokine (C-C motif) receptor-like 2 | 2.63 |
| Adora2a | adenosine A2a receptor | 2.55 |
| D330037A14Rik | RIKEN cDNA D330037A14 gene | 2.54 |
| Mki67 | antigen identified by monoclonal antibody Ki 67 | 2.49 |
| Mmp13 | matrix metalloproteinase 13 | 2.49 |
| Tnip1 | TNFAIP3 interacting protein 1 | 2.46 |
| Top1 | topoisomerase (DNA) I | 2.37 |
| F3 | coagulation factor III | 2.37 |
| Txnrd1 | thioredoxin reductase 1 | 2.36 |
| Gadd45b | growth arrest and DNA-damage-inducible 45 beta | 2.35 |
| Nfkb2 | nuclear factor of kappa light polypeptide gene enhancer in B-cells 2. p49/p100 | 2.34 |
| Sod2 | superoxide dismutase 2. mitochondrial | 2.33 |
| Nfkb1 | nuclear factor of kappa light chain gene enhancer in B-cells 1. p105 | 2.33 |
| Cav | caveolin. caveolae protein | 2.33 |
| Traf5 | Tnf receptor-associated factor 5 | 2.31 |
| Sqstm1 | sequestosome 1 | 2.30 |
| Trim13 | tripartite motif protein 13 | 2.27 |
| Cflar | CASP8 and FADD-like apoptosis regulator | 2.26 |
| Casp4 | caspase 4. apoptosis-related cysteine protease | 2.25 |
| Irf1 | interferon regulatory factor 1 | 2.23 |
| Egr2 | early growth response 2 | 2.23 |
| Ifi205 | interferon activated gene 205 | 2.21 |
| Slc2a1 | solute carrier family 2 (facilitated glucose transporter). member 1 | 2.21 |
| Nfkbib | nuclear factor of kappa light chain gene enhancer in B-cells inhibitor. beta | 2.17 |
| Erdr1-pending | erythroid differentiation regulator 1 | 2.15 |
| Slc30a1 | solute carrier family 30 (zinc transporter). member 1 | 2.14 |
| Dusp1 | dual specificity phosphatase 1 | 2.13 |
| Ell2 | elongation factor RNA polymerase II 2 | 2.10 |
| Siah1b | seven in absentia 1B | 2.06 |
| Gp38 | glycoprotein 38 | 2.05 |
| Spata13 | spermatogenesis associated 13 | 2.05 |
| Mox2 | antigen identified by monoclonal antibody MRC OX-2 | 2.02 |
| Ell2 | elongation factor RNA polymerase II 2 | 2.02 |
| Nfkbie | nuclear factor of kappa light polypeptide gene enhancer in B-cells inhibitor. epsilon | 2.01 |
| Plaur | urokinase plasminogen activator receptor | 2.01 |
| Kpna3 | karyopherin (importin) alpha 3 | 2.01 |
| Tgm2 | transglutaminase 2. C polypeptide | 2.01 |
| Mapkapk2 | MAP kinase-activated protein kinase 2 | 1.99 |
| Procr | protein C receptor. endothelial | 1.97 |
| Cd14 | CD14 antigen | 1.97 |
| Hspa1a | heat shock protein 1A | 1.96 |
| Stx3 | syntaxin 3 | 1.96 |
| Tes | testis derived transcript | 1.93 |
| Cdkn1a | cyclin-dependent kinase inhibitor 1A (P21) | 1.93 |
| Ptpre | protein tyrosine phosphatase. receptor type. E | 1.92 |
| Tubb3 | tubulin. beta 3 | 1.92 |
| Siat7d | sialyltransferase 7 ((alpha-N-acetylneuraminyl 2.3-betagalactosyl-1.3)-N-acetyl galactosaminide alpha-2.6-sialyltransferase) D | 1.92 |
| Itga5 | integrin alpha 5 (fibronectin receptor alpha) | 1.89 |
| E430019N21Rik | RIKEN cDNA E430019N21 gene | 1.88 |
| Gch | GTP cyclohydrolase 1 | 1.87 |
| Rrs1 | RRS1 ribosome biogenesis regulator homolog (S. cerevisiae) | 1.85 |
| Il17r | interleukin 17 receptor | 1.83 |
| Siat7d | sialyltransferase 7 ((alpha-N-acetylneuraminyl 2.3-betagalactosyl-1.3)-N-acetyl galactosaminide alpha-2.6-sialyltransferase) D | 1.83 |
| Ell2 | elongation factor RNA polymerase II 2 | 1.81 |
| Gclc | glutamate-cysteine ligase. catalytic subunit | 1.81 |
| Wfs1 | Wolfram syndrome 1 homolog (human) | 1.79 |
| Alas1 | aminolevulinic acid synthase 1 | 1.75 |
| Itgb4bp | integrin beta 4 binding protein | 1.75 |
| Tubb2 | tubulin. beta 2 | 1.75 |
| Btg1 | B-cell translocation gene 1. anti-proliferative | 1.75 |
| Ppan | peter pan homolog (Drosophila) | 1.75 |
| Wt1 | Wilms tumor homolog | 1.74 |
| Ccl2 | chemokine (C-C motif) ligand 2 | 1.71 |
| Psmd11 | proteasome (prosome. macropain) 26S subunit. non-ATPase. 11 | 1.71 |
| G3bp-pending | Ras-GTPase-activating protein SH3-domain binding protein | 1.67 |
| Numb | numb gene homolog (Drosophila) | 1.67 |
| Sfrs6 | splicing factor. arginine/serine-rich 6 | 1.67 |
| Rps6ka2 | ribosomal protein S6 kinase. polypeptide 2 | 1.66 |
| Hspa8 | heat shock protein 8 | 1.63 |
| Eno2 | enolase 2. gamma neuronal | 1.59 |
| Tpm1 | tropomyosin 1. alpha | 1.59 |
| Hspca | heat shock protein 1. alpha | 1.59 |
| Zfp36l1 | zinc finger protein 36. C3H type-like 1 | 0.21 |
| Adam19 | a disintegrin and metalloproteinase domain 19 (meltrin beta) | 0.26 |
| Mertk | c-mer proto-oncogene tyrosine kinase | 0.27 |
| Thbd | thrombomodulin | 0.27 |
| B3galt3 | UDP-Gal:betaGlcNAc beta 1.3-galactosyltransferase. polypeptide 3 | 0.28 |
| Mafb | v-maf musculoaponeurotic fibrosarcoma oncogene family. protein B (avian) | 0.28 |
| --- | Mus musculus Sestrin 1. mRNA (cDNA clone MGC:67135 IMAGE:6414521). complete cds | 0.29 |
| Frat2 | frequently rearranged in advanced T-cell lymphomas 2 | 0.29 |
| Nfic | nuclear factor I/C | 0.30 |
| Cnr2 | cannabinoid receptor 2 (macrophage) | 0.33 |
| Rgs2 | regulator of G-protein signaling 2 | 0.33 |
| Bpgm | 2.3-bisphosphoglycerate mutase | 0.35 |
| Pik3cd | phosphatidylinositol 3-kinase catalytic delta polypeptide | 0.35 |
| 4930422J18Rik | RIKEN cDNA 4930422J18 gene | 0.35 |
| Rassf2 | Ras association (RalGDS/AF-6) domain family 2 | 0.35 |
| Fli1 | Friend leukemia integration 1 | 0.36 |
| Icsbp1 | interferon consensus sequence binding protein 1 | 0.36 |
| 1110033A15Rik | RIKEN cDNA 1110033A15 gene | 0.37 |
| Sec14l1 | SEC14-like 1 (S. cerevisiae) | 0.37 |
| 9830126M18 | hypothetical protein 9830126M18 | 0.37 |
| Lpin1 | lipin 1 | 0.37 |
| Lyl1 | lymphoblastomic leukemia | 0.37 |
| 6330406L22Rik | RIKEN cDNA 6330406L22 gene | 0.37 |
| --- | Mus musculus transcribed sequence with weak similarity to protein ref:NP_001888.1 (H.sapiens) melanoma-associated chondroitin sulfate proteoglycan 4 [Homo sapiens] | 0.37 |
| 9830126M18 | hypothetical protein 9830126M18 | 0.39 |
| Nbr1 | neighbor of Brca1 gene 1 | 0.39 |
| 2610301B20Rik | RIKEN cDNA 2610301B20 gene | 0.40 |
| --- | Mus musculus transcribed sequences | 0.40 |
| Pdcd4 | programmed cell death 4 | 0.41 |
| AU020206 | expressed sequence AU020206 | 0.41 |
| 4930422J18Rik | RIKEN cDNA 4930422J18 gene | 0.42 |
| --- | Mus musculus cDNA clone MGC:37981 IMAGE:5137303. complete cds | 0.43 |
| Gmnn | geminin | 0.43 |
| Lyl1 | lymphoblastomic leukemia | 0.43 |
| AU043625 | expressed sequence AU043625 | 0.44 |
| Idb3 | inhibitor of DNA binding 3 | 0.44 |
| 9330177P20Rik | RIKEN cDNA 9330177P20 gene | 0.44 |
| 2510042P03Rik | RIKEN cDNA 2510042P03 gene | 0.44 |
| --- | --- | 0.45 |
| Hhex | hematopoietically expressed homeobox | 0.45 |
| Notch1 | Notch gene homolog 1 (Drosophila) | 0.46 |
| Ptger2 | prostaglandin E receptor 2 (subtype EP2) | 0.46 |
| Thbd | thrombomodulin | 0.46 |
| Rasa3 | RAS p21 protein activator 3 | 0.47 |
| E130315B21Rik | RIKEN cDNA E130315B21 gene | 0.48 |
| Cbx4 | chromobox homolog 4 (Drosophila Pc class) | 0.48 |
| H1f0 | H1 histone family. member 0 | 0.48 |
| 1700065A05Rik | RIKEN cDNA 1700065A05 gene | 0.48 |
| BC039093 | cDNA sequence BC039093 | 0.50 |
| Waspip | Wiskott-Aldrich syndrome protein interacting protein | 0.50 |
| Hist1h1c | histone 1. H1c | 0.50 |
| D4Wsu53e | DNA segment. Chr 4. Wayne State University 53. expressed | 0.50 |
| Zfp118 | Zinc finger protein 118 | 0.50 |
| 1110018O08Rik | RIKEN cDNA 1110018O08 gene | 0.51 |
| Gab1 | growth factor receptor bound protein 2-associated protein 1 | 0.51 |
| Fbxo8 | F-box only protein 8 | 0.51 |
| 6720457D02Rik | RIKEN cDNA 6720457D02 gene | 0.51 |
| D6Ertd32e | DNA segment. Chr 6. ERATO Doi 32. expressed | 0.51 |
| 4833420G17Rik | RIKEN cDNA 4833420G17 gene | 0.51 |
| Gpcr25 | G-protein coupled receptor 25 | 0.51 |
| Snrk | SNF related kinase | 0.51 |
| Rbl2 | retinoblastoma-like 2 | 0.51 |
| AI661017 | expressed sequence AI661017 | 0.52 |
| Osbpl11 | oxysterol binding protein-like 11 | 0.52 |
| Cerk | ceramide kinase | 0.53 |
| 2410003A14Rik | RIKEN cDNA 2410003A14 gene | 0.53 |
| Tcf4 | transcription factor 4 | 0.53 |
| LOC269796 | hypothetical protein LOC269796 | 0.53 |
| Ccng2 | cyclin G2 | 0.53 |
| 0610043B10Rik | RIKEN cDNA 0610043B10 gene | 0.53 |
| Madh6 | MAD homolog 6 (Drosophila) | 0.54 |
| 2310005P05Rik | RIKEN cDNA 2310005P05 gene | 0.54 |
| 1110048B16Rik | RIKEN cDNA 1110048B16 gene | 0.54 |
| C130065N10Rik | RIKEN cDNA C130065N10 gene | 0.54 |
| Stk38 | serine/threonine kinase 38 | 0.54 |
| Zdhhc14 | zinc finger. DHHC domain containing 14 | 0.54 |
| Rpa3 | replication protein A3 | 0.54 |
| Tec | cytoplasmic tyrosine kinase. Dscr28C related (Drosophila) | 0.54 |
| Mad4 | Max dimerization protein 4 | 0.54 |
| Inpp5d | inositol polyphosphate-5-phosphatase D | 0.54 |
| 2810429C13Rik | RIKEN cDNA 2810429C13 gene | 0.55 |
| 1110054N06Rik | RIKEN cDNA 1110054N06 gene | 0.55 |
| Sipa1 | signal-induced proliferation associated gene 1 | 0.55 |
| BC058408 | cDNa sequence BC058408 | 0.55 |
| Rassf5 | Ras association (RalGDS/AF-6) domain family 5 | 0.55 |
| 1110018J12Rik | RIKEN cDNA 1110018J12 gene | 0.55 |
| Dscr1l2 | Down syndrome critical region gene 1-like 2 | 0.55 |
| Eif2ak3 | eukaryotic translation initiation factor 2 alpha kinase 3 | 0.55 |
| Il6ra | interleukin 6 receptor. alpha | 0.55 |
| C030048H19Rik | RIKEN cDNA C030048H19 gene | 0.56 |
| Zfp90 | zinc finger protein 90 | 0.56 |
| Bcl2l11 | BCL2-like 11 (apoptosis facilitator) | 0.56 |
| 5330434F23Rik | RIKEN cDNA 5330434F23 gene | 0.56 |
| Xpa | xeroderma pigmentosum. complementation group A | 0.56 |
| Mbtd1 | mbt domain containing 1 | 0.56 |
| Tk2 | thymidine kinase 2. mitochondrial | 0.56 |
| Ngrn-pending | neugrin | 0.56 |
| Pou2f1 | POU domain. class 2. transcription factor 1 | 0.57 |
| Gtpi-pending | interferon-g induced GTPase | 0.57 |
| Atp7a | ATPase. Cu++ transporting. alpha polypeptide | 0.57 |
| Casp2 | caspase 2 | 0.58 |
| Ssx2ip | synovial sarcoma. X breakpoint 2 interacting protein | 0.58 |
| --- | Mus musculus cDNA clone MGC:59570 IMAGE:6506619. complete cds | 0.58 |
| C330008I15Rik | RIKEN cDNA C330008I15 gene | 0.58 |
| Mbp | myelin basic protein | 0.58 |
| Hmgb2 | high mobility group box 2 | 0.58 |
| --- | --- | 0.58 |
| Helb | helicase (DNA) B | 0.59 |
| Ulk1 | Unc-51 like kinase 1 (C. elegans) | 0.60 |
| Vrk3 | vaccinia related kinase 3 | 0.60 |
| Clcn3 | chloride channel 3 | 0.60 |
| Rab3d | RAB3D. member RAS oncogene family | 0.60 |
| Epb4.1 | erythrocyte protein band 4.1 | 0.60 |
| Ptk9l | PTK9 protein typrotein tyrosine kinase 9-like (A6-related protein) | 0.62 |
| 1110007A06Rik | RIKEN cDNA 1110007A06 gene | 0.62 |
| A130052D22 | hypothetical protein A130052D22 | 0.63 |
| Gt(ROSA)26asSor | gene trap ROSA 26 antisense. Philippe Soriano | 0.63 |
| Tep1 | telomerase associated protein 1 | 0.63 |
| D19Ertd144e | DNA segment. Chr 19. ERATO Doi 144. expressed | 0.63 |
| Adprtl2 | ADP-ribosyltransferase (NAD+; poly(ADP-ribose) polymerase)-like 2 | 0.63 |
| Echs1 | enoyl Coenzyme A hydratase. short chain. 1. mitochondrial | 0.63 |
| Sema4a | sema domain. immunoglobulin domain (Ig). transmembrane domain (TM) and short cytoplasmic domain. (semaphorin) 4A | 0.63 |
| Polr2e | polymerase (RNA) II (DNA directed) polypeptide E | 0.65 |
| Siat8d | sialyltransferase 8 (alpha-2. 8-sialyltransferase) D | 0.65 |
| Ppp1cc | protein phosphatase 1. catalytic subunit. gamma isoform | 0.65 |
| Casp9 | caspase 9 | 0.65 |
| D10Wsu52e | DNA segment. Chr 10. Wayne State University 52. expressed | 0.66 |

# Supplementary Table 2

| Supplementary Table 2: Genes regulated in 4h *Leishmania major* infected granuloma macrophages from BALB/c mice | | | |
| --- | --- | --- | --- |
| Gene name | Description | n-fold | |
| Gem | GTP binding protein (gene overexpressed in skeletal muscle) | | 12.50 |
| Cxcl2 | chemokine (C-X-C motif) ligand 2 | | 12.05 |
| Cxcl1 | chemokine (C-X-C motif) ligand 1 | | 10.71 |
| Cxcl1 | chemokine (C-X-C motif) ligand 1 | | 9.90 |
| Ccl4 | chemokine (C-C motif) ligand 4 | | 8.67 |
| Tnf | tumor necrosis factor | | 8.16 |
| Traf1 | Tnf receptor-associated factor 1 | | 8.14 |
| Il1a | interleukin 1 alpha | | 6.12 |
| Cd83 | CD83 antigen | | 5.04 |
| Ahr | aryl-hydrocarbon receptor | | 4.58 |
| Ets2 | E26 avian leukemia oncogene 2, 3' domain | | 4.28 |
| Fosl1 | fos-like antigen 1 | | 4.23 |
| Ccl3 | chemokine (C-C motif) ligand 3 | | 4.18 |
| Npn3 | neoplastic progression 3 | | 3.79 |
| Snk | serum-inducible kinase | | 3.60 |
| Egr2 | early growth response 2 | | 3.59 |
| 1190002H23Rik | RIKEN cDNA 1190002H23 gene | | 3.57 |
| A230108E06 | hypothetical protein A230108E06 | | 3.52 |
| Sphk1 | sphingosine kinase 1 | | 3.30 |
| Irg1 | immunoresponsive gene 1 | | 3.22 |
| Il1rn | interleukin 1 receptor antagonist | | 3.21 |
| Ptges | prostaglandin E synthase | | 3.00 |
| Ralgds | ral guanine nucleotide dissociation stimulator | | 2.94 |
| Ifrd1 | interferon-related developmental regulator 1 | | 2.85 |
| Gadd45b | growth arrest and DNA-damage-inducible 45 beta | | 2.78 |
| Mlp | MARCKS-like protein | | 2.77 |
| Irg1 | immunoresponsive gene 1 | | 2.67 |
| --- | --- | | 2.63 |
| Ugcg | UDP-glucose ceramide glucosyltransferase | | 2.49 |
| Gadd45a | growth arrest and DNA-damage-inducible 45 alpha | | 2.47 |
| Pcdh7 | protocadherin 7 | | 2.45 |
| Txnrd1 | thioredoxin reductase 1 | | 2.45 |
| Slc2a1 | solute carrier family 2 (facilitated glucose transporter), member 1 | | 2.45 |
| Slc11a2 | solute carrier family 11 (proton-coupled divalent metal ion transporters), member 2 | | 2.43 |
| --- | Mus musculus transcribed sequence with weak similarity to protein ref:NP_081764.1 (M.musculus) RIKEN cDNA 5730493B19 [Mus musculus] | | 2.41 |
| Plau | plasminogen activator, urokinase | | 2.40 |
| Myd116 | myeloid differentiation primary response gene 116 | | 2.38 |
| Adora2a | adenosine A2a receptor | | 2.31 |
| 0610012A05Rik | RIKEN cDNA 0610012A05 gene | | 2.27 |
| Gadd45b | growth arrest and DNA-damage-inducible 45 beta | | 2.27 |
| Clecsf9 | C-type (calcium dependent, carbohydrate recognition domain) lectin, superfamily member 9 | | 2.26 |
| Socs3 | suppressor of cytokine signaling 3 | | 2.26 |
| Atf3 | activating transcription factor 3 | | 2.22 |
| AI462446 | expressed sequence AI462446 | | 2.18 |
| Trim13 | tripartite motif protein 13 | | 2.12 |
| Eno2 | enolase 2, gamma neuronal | | 2.09 |
| --- | --- | | 2.07 |
| Bhlhb2 | basic helix-loop-helix domain containing, class B2 | | 2.01 |
| Siat7d | sialyltransferase 7 ((alpha-N-acetylneuraminyl 2,3-betagalactosyl-1,3)-N-acetyl galactosaminide alpha-2,6-sialyltransferase) D | | 2.00 |
| Ampd3 | AMP deaminase 3 | | 1.96 |
| Cd14 | CD14 antigen | | 1.95 |
| C230060M08Rik | RIKEN cDNA C230060M08 gene | | 1.94 |
| Siat7d | sialyltransferase 7 ((alpha-N-acetylneuraminyl 2,3-betagalactosyl-1,3)-N-acetyl galactosaminide alpha-2,6-sialyltransferase) D | | 1.91 |
| Zfp216 | zinc finger protein 216 | | 1.91 |
| Rgl1 | ral guanine nucleotide dissociation stimulator,-like 1 | | 1.88 |
| Btg1 | B-cell translocation gene 1, anti-proliferative | | 1.87 |
| Hspca | heat shock protein 1, alpha | | 1.83 |
| Atf3 | activating transcription factor 3 | | 1.80 |
| Stx3 | syntaxin 3 | | 1.79 |
| Sfrs6 | splicing factor, arginine/serine-rich 6 | | 1.79 |
| Sdc1 | syndecan 1 | | 1.76 |
| Nfkb1 | nuclear factor of kappa light chain gene enhancer in B-cells 1, p105 | | 1.75 |
| 2310057H16Rik | RIKEN cDNA 2310057H16 gene | | 1.75 |
| Mdm2 | transformed mouse 3T3 cell double minute 2 | | 1.72 |
| Tardbp | TAR DNA binding protein | | 1.71 |
| Tollip | toll interacting protein | | 1.70 |
| Serpinb9 | serine (or cysteine) proteinase inhibitor, clade B, member 9 | | 1.66 |
| Eif5 | eukaryotic translation initiation factor 5 | | 1.63 |
| Tank | TRAF family member-associated Nf-kappa B activator | | 1.52 |
| BC042396 | cDNA sequence BC042396 | | 0.27 |
| --- | Mus musculus Sestrin 1, mRNA (cDNA clone MGC:67135 IMAGE:6414521), complete cds | | 0.28 |
| Dusp6 | dual specificity phosphatase 6 | | 0.28 |
| 4930422J18Rik | RIKEN cDNA 4930422J18 gene | | 0.29 |
| Fli1 | Friend leukemia integration 1 | | 0.33 |
| Mafb | v-maf musculoaponeurotic fibrosarcoma oncogene family, protein B (avian) | | 0.35 |
| Zfp36l1 | zinc finger protein 36, C3H type-like 1 | | 0.35 |
| Pik3cd | phosphatidylinositol 3-kinase catalytic delta polypeptide | | 0.37 |
| Lyl1 | lymphoblastomic leukemia | | 0.37 |
| Rassf2 | Ras association (RalGDS/AF-6) domain family 2 | | 0.38 |
| 4930422J18Rik | RIKEN cDNA 4930422J18 gene | | 0.38 |
| --- | Mus musculus transcribed sequences | | 0.39 |
| Ccr2 | chemokine (C-C) receptor 2 | | 0.39 |
| Smarcf1 | SWI/SNF related, matrix associated, actin dependent regulator of chromatin, subfamily f, member 1 | | 0.39 |
| Hhex | hematopoietically expressed homeobox | | 0.39 |
| Thbd | thrombomodulin | | 0.40 |
| Lyl1 | lymphoblastomic leukemia | | 0.40 |
| Sec14l1 | SEC14-like 1 (S. cerevisiae) | | 0.41 |
| Ptger2 | prostaglandin E receptor 2 (subtype EP2) | | 0.41 |
| Bpgm | 2,3-bisphosphoglycerate mutase | | 0.43 |
| Mertk | c-mer proto-oncogene tyrosine kinase | | 0.43 |
| Rin2 | Ras and Rab interactor 2 | | 0.43 |
| E130315B21Rik | RIKEN cDNA E130315B21 gene | | 0.44 |
| Pr1 | protein distantly related to to the gamma subunit family | | 0.44 |
| Gpcr25 | G-protein coupled receptor 25 | | 0.44 |
| 9830126M18 | hypothetical protein 9830126M18 | | 0.44 |
| 9830126M18 | hypothetical protein 9830126M18 | | 0.45 |
| Notch1 | Notch gene homolog 1 (Drosophila) | | 0.46 |
| Gmnn | geminin | | 0.47 |
| 0610013D04Rik | RIKEN cDNA 0610013D04 gene | | 0.47 |
| Sps2 | selenophosphate synthetase 2 | | 0.48 |
| Notch1 | Notch gene homolog 1 (Drosophila) | | 0.48 |
| 1110033A15Rik | RIKEN cDNA 1110033A15 gene | | 0.49 |
| Zfp118 | Zinc finger protein 118 | | 0.49 |
| Klf13 | Kruppel-like factor 13 | | 0.50 |
| Atp7a | ATPase, Cu++ transporting, alpha polypeptide | | 0.50 |
| Rbl2 | retinoblastoma-like 2 | | 0.51 |
| AU043625 | expressed sequence AU043625 | | 0.51 |
| 1110018J12Rik | RIKEN cDNA 1110018J12 gene | | 0.51 |
| Pcyox1 | prenylcysteine oxidase 1 | | 0.52 |
| --- | --- | | 0.52 |
| Zfp161 | zinc finger protein 161 | | 0.52 |
| Dri1 | dead ringer homolog 1 (Drosophila) | | 0.52 |
| Rgs2 | regulator of G-protein signaling 2 | | 0.53 |
| Cebpd | CCAAT/enhancer binding protein (C/EBP), delta | | 0.53 |
| H1f0 | H1 histone family, member 0 | | 0.53 |
| Cbx4 | chromobox homolog 4 (Drosophila Pc class) | | 0.53 |
| Ssx2ip | synovial sarcoma, X breakpoint 2 interacting protein | | 0.54 |
| Gab1 | growth factor receptor bound protein 2-associated protein 1 | | 0.54 |
| C030048H19Rik | RIKEN cDNA C030048H19 gene | | 0.54 |
| Ptk9l | PTK9 protein typrotein tyrosine kinase 9-like (A6-related protein) | | 0.55 |
| BC039093 | cDNA sequence BC039093 | | 0.55 |
| 3632413B07Rik | RIKEN cDNA 3632413B07 gene | | 0.55 |
| Ski | Sloan-Kettering viral oncogene homolog | | 0.55 |
| Echs1 | enoyl Coenzyme A hydratase, short chain, 1, mitochondrial | | 0.55 |
| BC023892 | cDNA sequence BC023892 | | 0.56 |
| --- | Mus musculus mRNA for mKIAA1523 protein | | 0.56 |
| Eed | embryonic ectoderm development | | 0.56 |
| Sipa1 | signal-induced proliferation associated gene 1 | | 0.56 |
| Tec | cytoplasmic tyrosine kinase, Dscr28C related (Drosophila) | | 0.56 |
| D6Ertd32e | DNA segment, Chr 6, ERATO Doi 32, expressed | | 0.56 |
| Icsbp1 | interferon consensus sequence binding protein 1 | | 0.56 |
| Bcl2l11 | BCL2-like 11 (apoptosis facilitator) | | 0.56 |
| 2810407K09Rik | RIKEN cDNA 2810407K09 gene | | 0.57 |
| 2210023F24Rik | RIKEN cDNA 2210023F24 gene | | 0.57 |
| 0610043B10Rik | RIKEN cDNA 0610043B10 gene | | 0.58 |
| AU020206 | expressed sequence AU020206 | | 0.58 |
| 2610319K07Rik | RIKEN cDNA 2610319K07 gene | | 0.58 |
| 1500041O16Rik | RIKEN cDNA 1500041O16 gene | | 0.58 |
| Zdhhc14 | zinc finger, DHHC domain containing 14 | | 0.58 |
| D19Ertd144e | DNA segment, Chr 19, ERATO Doi 144, expressed | | 0.58 |
| Cpo | coproporphyrinogen oxidase | | 0.59 |
| Cpo | coproporphyrinogen oxidase | | 0.59 |
| AA960287 | expressed sequence AA960287 | | 0.60 |
| 2410003A14Rik | RIKEN cDNA 2410003A14 gene | | 0.60 |
| Bysl | bystin-like | | 0.60 |
| Pias3 | protein inhibitor of activated STAT 3 | | 0.60 |
| D1Ertd622e | DNA segment, Chr 1, ERATO Doi 622, expressed | | 0.61 |
| Clast3-pending | CD40 ligand-activated specific transcript 3 | | 0.61 |
| Tcf4 | transcription factor 4 | | 0.62 |
| Pura | purine rich element binding protein A | | 0.62 |
| Hsd17b4 | hydroxysteroid (17-beta) dehydrogenase 4 | | 0.62 |
| D930014A20Rik | RIKEN cDNA D930014A20 gene | | 0.62 |
| Snrk | SNF related kinase | | 0.62 |
| Rnase4 | ribonuclease, RNase A family 4 | | 0.62 |
| C80913 | expressed sequence C80913 | | 0.63 |
| Fut8 | fucosyltransferase 8 | | 0.63 |
| Waspip | Wiskott-Aldrich syndrome protein interacting protein | | 0.63 |
| D11Ertd603e | DNA segment, Chr 11, ERATO Doi 603, expressed | | 0.63 |
| Fbxo8 | F-box only protein 8 | | 0.65 |
| Cpt1a | carnitine palmitoyltransferase 1, liver | | 0.65 |
