# Supplementary figures and images for: Aryl Hydrocarbon Receptor-Signaling Regulates Early Leishmania major-Induced Cytokine Expression
Source: Front Immunol. 2019 Oct 15;10:2442. doi: 10.3389/fimmu.2019.02442 (PMC6843081; doi:10.3389/fimmu.2019.02442)

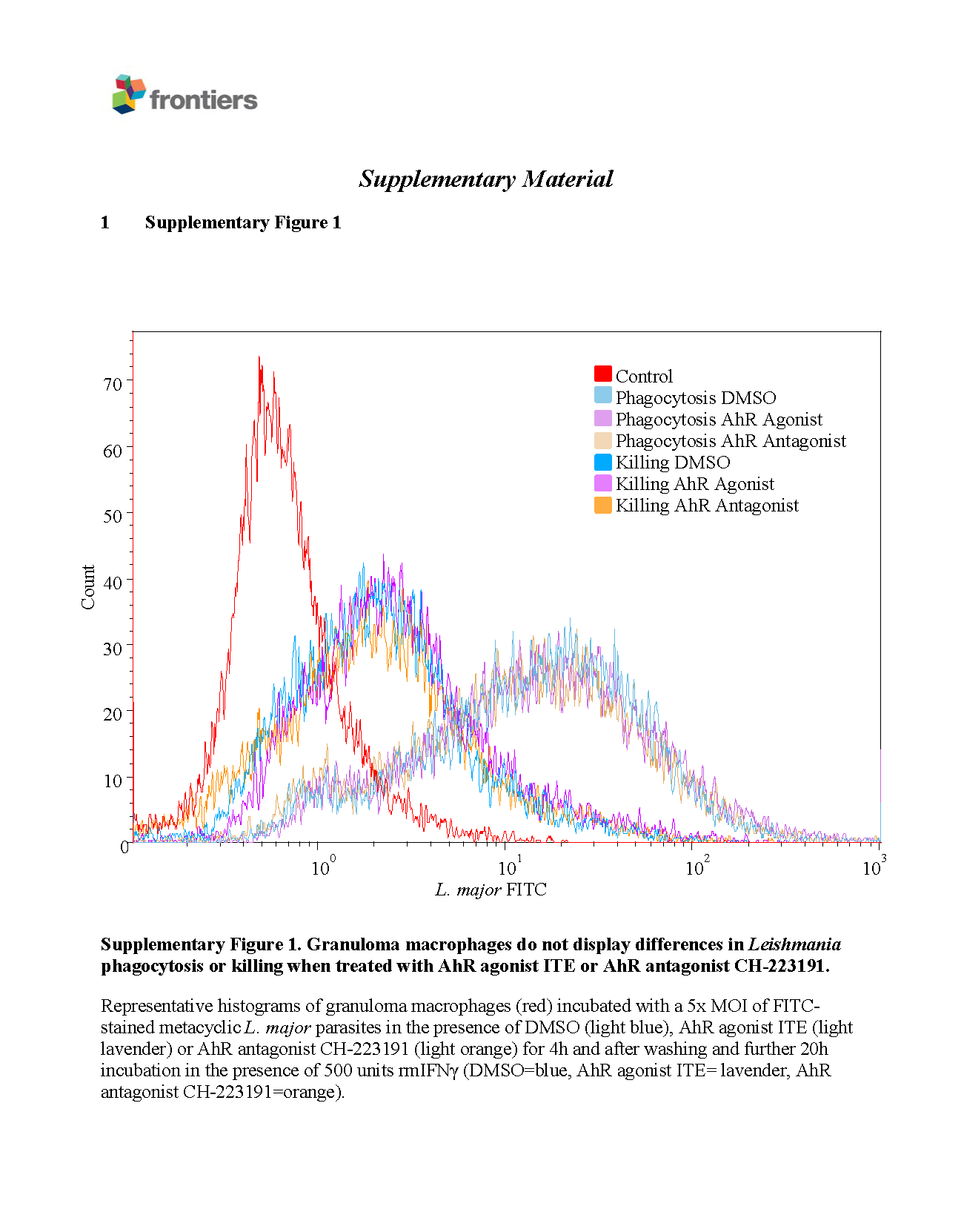

Supplement: Supplementary file 2 [file Image_1.TIFF]
